# Biomechanical evaluation of a novel wheelchair backrest for elderly people

**DOI:** 10.1186/s12938-015-0008-6

**Published:** 2015-02-21

**Authors:** Chun-Ting Li, Chih-Hsien Chen, Yen-Nien Chen, Chih-Han Chang, Kuen-Horng Tsai

**Affiliations:** Department of BioMedical Engineering, National Cheng Kung University, No.1, University Road, Tainan City, 701 Taiwan; Department of Orthopaedic Surgery, Tainan Municipal Hospital, No.670, Chongde Rd., East Dist., Tainan City, 701 Taiwan; Graduate Institute of Mechatronic System Engineering, National University of Tainan, 33, Sec. 2, Shu-Lin St., Tainan, 70005 Taiwan

**Keywords:** Back pain, Backrest, Sitting posture, Interface pressure, Discomfort, and Wheelchair

## Abstract

**Background:**

Back pain is a common complication of wheelchair-bound elderly people. Seating system is a key factor that influences spinal curvature, back muscle activation, interface pressure, and comfortability. A seating system can maintain lumbar lordosis, lower back muscle activity, and decrease ischial tuberosities pressure, which reduces spinal load and directly influences sitting comfort. Our previous study has confirmed that backward thoracic support showed a relatively higher lumbar lordosis and lower back muscle activity. This study intends to evaluate the influence of backward thoracic support on interface pressure and subjective discomfort.

**Methods:**

In this study, 18 elderly men were recruited to participate in a random comparison involving 4 sitting postures. These postures comprised relaxed slouching, flat back support, prominent lumbar support, and backward thoracic support sitting. All parameters, including interface pressure (total contact area, average pressure, and peak pressure on backrest and seat) and subjective discomfort (upper-back, mid-back, lower-back, buttocks, and thighs) were measured and compared.

**Results:**

The results showed that compared with other sitting postures, backward thoracic support sitting significantly reduced average pressure and peak pressure on seat and increased average pressure and peak pressure on backrest. Concurrently, subjective discomfort in the upper-back, mid-back, lower-back, and buttocks were reduced.

**Conclusions:**

The results confirmed that backward thoracic support can maintain favorable wheelchair sitting posture, thereby preventing or reducing the risks of back pain. However, this study was no evaluations on shear forces on butts and neck postures. Future studies investigating shear forces on butts and neck postures are required.

## Introduction

Back pain is a common complication among wheelchair users [1,2]. Clinical observations have shown that wheelchair-bound elderly people often present a slouching sitting posture [2]. It produces a flexion-relaxation phenomenon (FRP) in which the trunk stabilizers engaged in changes from active (muscles and tendons) to passive (intervertebral discs and ligaments) structures [3]. The FRP generates stress and causes metabolite accumulations in intervertebral discs, exposing wheelchair users to increased risks of degeneration and pain over time [4,5]. Furthermore, a continuous static load within the ligaments of the lumbar spine may cause spasm and hyperexcitability in vivo feline model [6]. Previous studies have indicated that maintaining lumbar lordosis, decreasing back muscle activity, or diminishing the pressure on ischial tuberosities (IT) facilitates the reduction of spinal load [4,5,7,8]. For wheelchair-bound elderly people, selecting a suitable wheelchair seating system is especially crucial and directly influences the spinal curvature, back muscle activation, interface pressure, and comfortability of sitting posture [9-13].

Wheelchair seating systems can be divided into dynamic and static systems. The dynamic system primarily comprised backrests and seat cushions that feature various combinations (i.e., array-type, partitioned-type) and driving structures (i.e., machinery-type, airbag-type), body postures were changed periodically to prevent prolonged pressure exertion in one region (power-assisted). The static system primarily alleviates pressure by using backrests and seat cushions composed of various materials or shapes (without power-assist). Numerous studies have produced satisfactory results regarding dynamic systems [11-13]. However, because of medical cost and personal income considerations, dynamic systems are rarely used in hospitals. Instead, inexpensive, simple, and nonelectrical static systems are employed. Presently, the standard sling seat and back upholstery, the most commonly used static system, has a limited effect on maintaining optimal posture [2]. Previous studies have indicated that a reclined backrest and lumbar support can help reduce IT stress and lumbar load [14,15]. However, when a backrest is reclined, daily living functions such as observing, eating, reaching for objects, or pushing the wheelchair are influenced [15,16]. Additionally, elderly people on a reclined backrest are at risk of poor sitting postures because of forward sliding [15]. Lumbar support can maintain lumbar lordosis, thereby reducing stress on intervertebral discs [4,5]. However, in contrast to lumbar kyphosis, lumbar lordosis is accompanied by anterior pelvic tilt in lumbar-pelvic rhythm [17]. Additionally, pelvic tilt is related to hamstring tightness. Anterior pelvic tilt increases hamstring tightness, whereas the hamstring is loosened in posterior pelvic tilt [18,19]. Thus, to enable sufficient lumbar lordosis, anterior pelvic tilt and hamstring tightness are unavoidable, which subsequently influence the comfortability of sitting posture maintenance. Although numerous studies on wheelchair seating systems have been conducted, it seems that the aspect of aforementioned problems can still be further improved [20,21].

**Figure 1:**
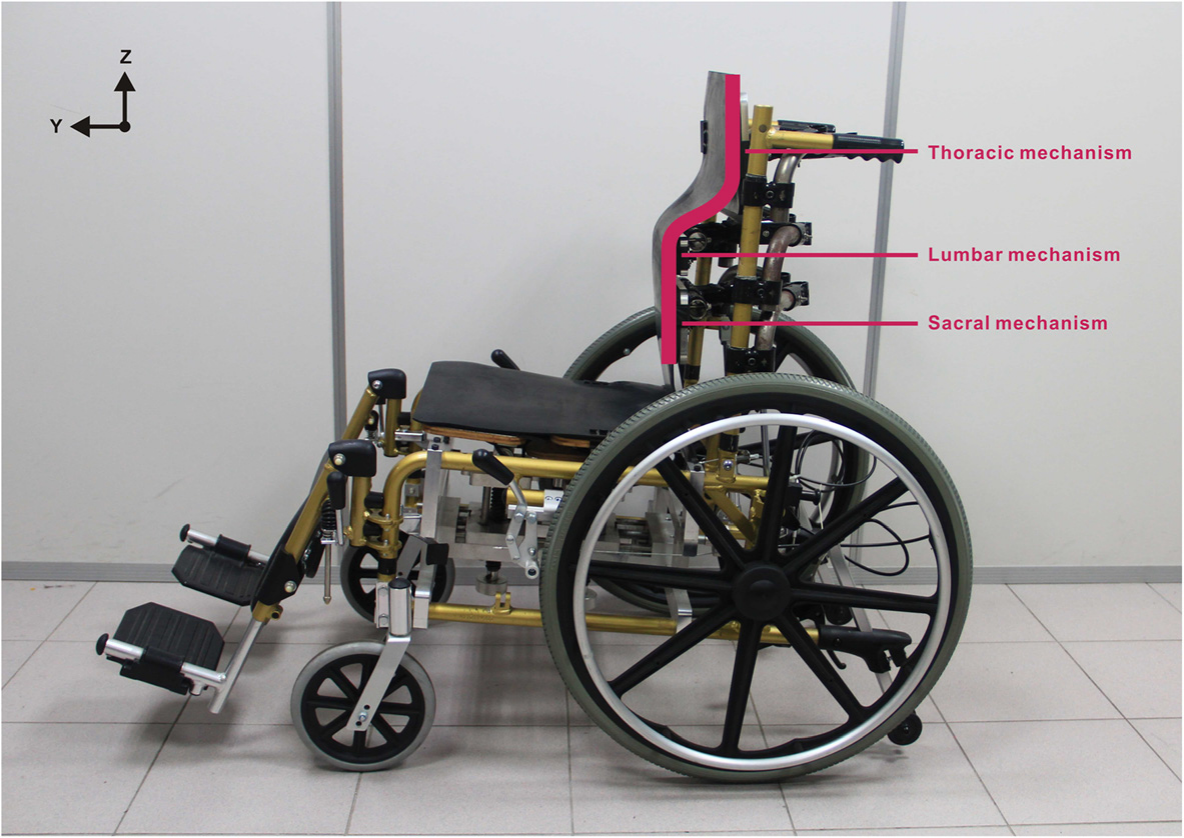
**Experimental wheelchair.** The picture shows the actual experimental wheelchair, the backrest was divided into 3 parts, comprising thoracic, lumbar, and sacral adjustment mechanisms. Each mechanism is capable of rotating around the X-axis and translating about the Y-axis and the Z-axis. The seat height, seat depth, footrest length, and footrest angles can all be adjusted. The current setting is backward thoracic support in the sagittal plane.

Our previous study has introduced a novel wheelchair seating system concept: backward thoracic support (Figure 1) [22], it was confirmed that this backward thoracic support concept showed a relatively higher lumbar lordosis, neutral pelvic tilt (i.e., on the sagittal plane, the horizontal line connecting the two points of posterior superior iliac spines (PSISs) and anterior superior iliac spines (ASISs) on the pelvis is the neutral pelvic tilt [23]), and lower back muscle activity. However, this finding was only about the relationships between spinal curvature and back muscle activation, which did not provide the relationships between interface pressure and subjective discomfort on the backward thoracic support. Therefore, the purpose of this study was to evaluate the influence of backward thoracic support sitting (BTS) on elderly people regarding interface pressure and subjective discomfort. The hypothesis was as follows: When the thoracic mechanism is backward adjusted, it results in redistributing interface pressure to reduce IT stress and reducing subjective discomfort in the back and buttocks. This study also compared BTS with common wheelchair sitting postures, which include relaxed slouching sitting (RSS), flat back support sitting (FBS), and prominent lumbar support sitting (PLS).

## Methods

### Participants

In this study, participants had to be elderly adults (aged 65 years and over) who had no any known spinal pathology or musculoskeletal disorders. All the participants read and signed an informed consent form, which explained the objectives of the study and the experimental protocol. This study was approved by the Institutional Review Board of National Cheng Kung University Hospital.

### Experimental protocol

**Figure 2:**
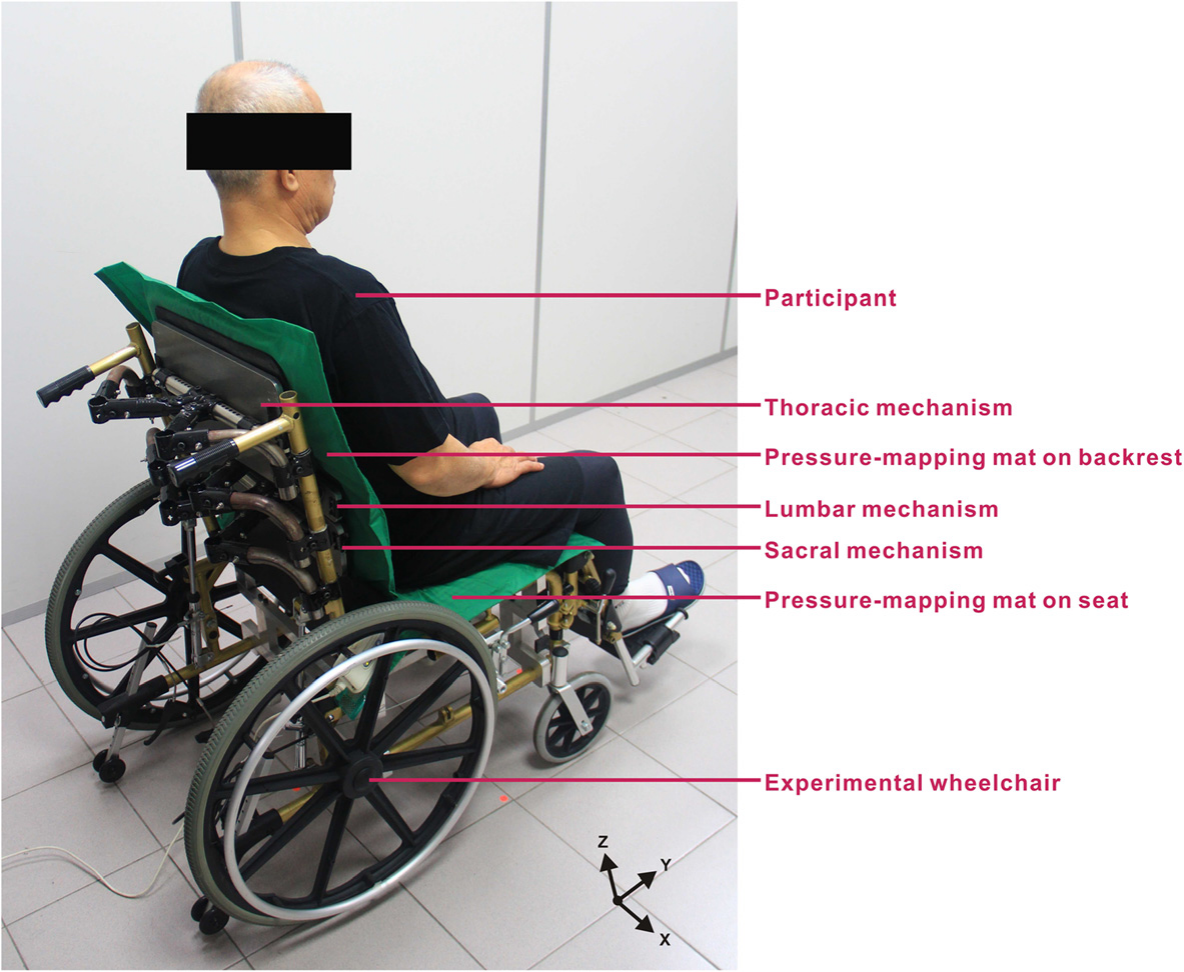
**Experimental setup.** The picture shows the experimental setup with participant, experimental wheelchair, and pressure-mapping mats. Two pressure-mapping mats were placed over the surface of the backrest and the seat to measure pressure distribution.

Prior to data collection, the depth and height of experimental wheelchair seat were adjusted to accommodate the participants’ popliteal fossa. Each participant was then transferred to the experimental wheelchair with their upper bodies leaning against the backrest, arms relaxed on both sides of the body, thighs kept parallel to the ground, feet firmly positioned on the footpad at shoulders’ width, and eyes leveled toward the front (Figure 2). Subsequently, the participants completed the four posture experiments in a random order. Regarding interface pressure measurements, data were collected for 5 s when the participant maintained a stable posture; participants were then requested to stand up and move around for 1 min before changing to the next posture. Regarding subjective discomfort evaluations, the participants were asked to remain still for 15 min in each posture. Before changing to the next posture, the participants were allowed 5 min breaks, during which they answered the subjective discomfort evaluation for each posture.

### Wheelchair set-up

Figure 1 shows the unique experimental wheelchair designed in this study [22]. The backrest was divided into 3 parts, comprising thoracic, lumbar, and sacral adjustment mechanisms. Each mechanism is capable of rotating around the X-axis and translating about the Y-axis and the Z-axis. The seat height, seat depth, footrest length, and footrest angles can all be adjusted. In addition, the backrest and seat cushion were each installed with a 2.5 cm-thick foam to reduce the discomfort caused by the concentrated stress at the skin contact area.

### Postures

**Figure 3:**
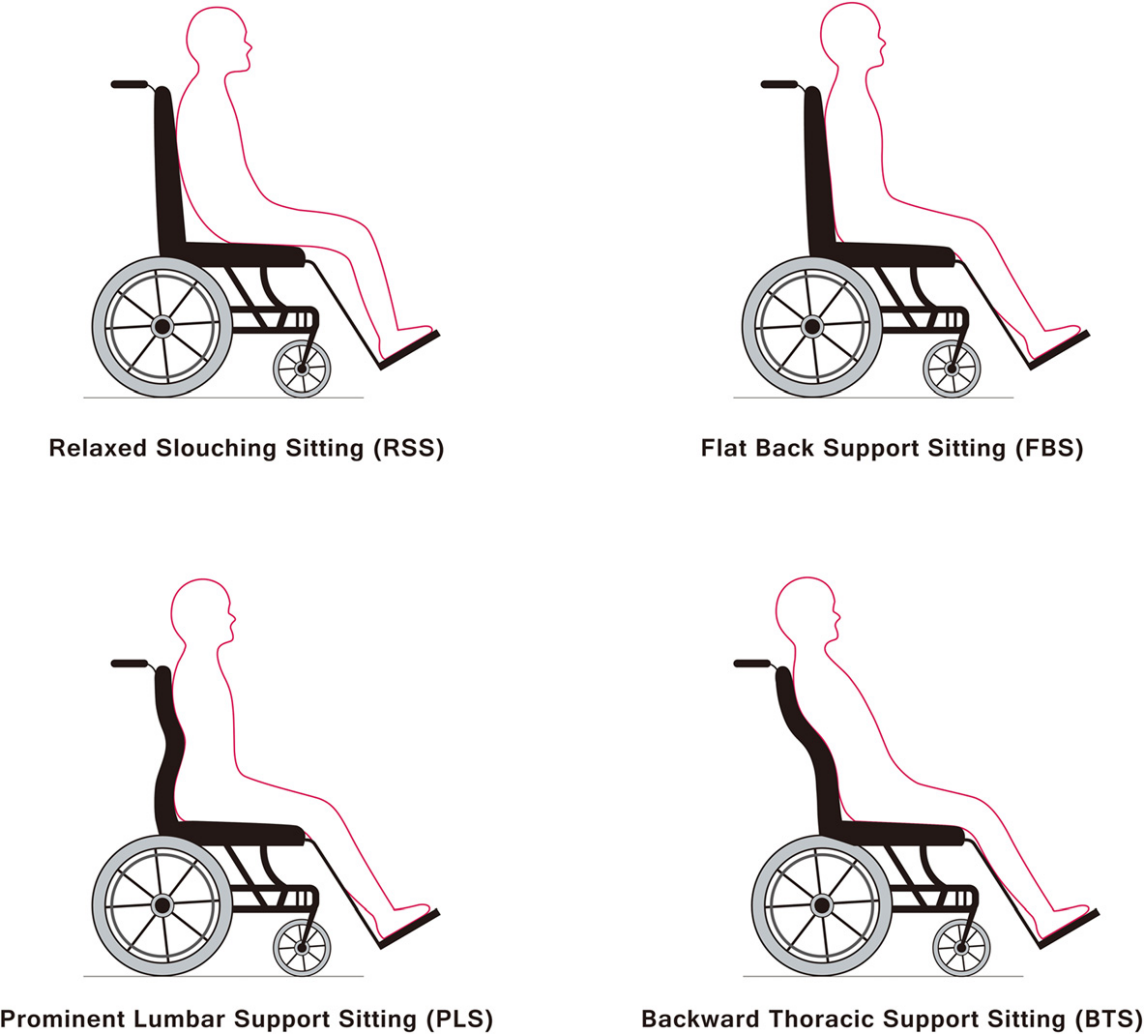
**Four different sitting postures.** Include relaxed slouching sitting (RSS), flat back support sitting (FBS), prominent lumbar support sitting (PLS), and backward thoracic support sitting (BTS).

In this study, we compared 4 sitting postures, RSS, FBS, PLS, and BTS (Figure 3). These sitting postures are described as follows:

RSS: The backrest of experimental wheelchair is kept flat; the pelvis of participant is positioned in the midsection of the seat cushion, allowing it to significantly tilt posteriorly; and the torso of participant presents a C-curve and rests against the backrest [9].

FBS: The backrest of experimental wheelchair is kept flat; the pelvis of participant is pushed back and the torso of participant leans against the backrest [9].

PLS: The lumbar adjustment mechanism of experimental wheelchair was configured with a 4-cm protrusion. At the L3 vertebra of participant, the pelvis of participant is moved as far back as possible with the torso of participant leaning against the backrest [24,25].

BTS: The thoracic adjustment mechanism of experimental wheelchair was retracted by 8 cm. At the T7-T12 vertebra of participant, the pelvis of participant was moved back as far as possible with the torso of participant leaning against the backrest [22].

### Data recording and analysis

Interface pressure measurements: Two pressure-mapping mats (Body Pressure Measurement System; Tekscan Inc, South Boston, Massachusetts, USA) were placed over the surface of the backrest and the seat to measure pressure distribution. The mats comprised 2016 (48 × 42) measuring cells. Each measuring cell had a dimension of 10.16 × 10.16 mm^2^. The sampling frequency for pressure mapping was set to 100 Hz. The total contact area (TCA), average pressure (AP), and peak pressure (PP) on both the whole backrest (TCA_BACK_, AP_BACK_, and PP_BACK_) and the entire seat (TCA_SEAT_, AP_SEAT_, and PP_SEAT_) were calculated.

Subjective discomfort evaluations: We used the body part discomfort scale designed by Corlett and Bishop to determine the subjective discomfort as perceived by the participants [26]. In this questionnaire, the analyzed body parts were divided into the upper-back, mid-back, lower-back, buttocks, and thighs. The question items for each body part were evaluated using a scale of 6 levels (0–5) in which 0 represents no discomfort and 5 represents extreme conceivable discomfort.

### Statistical analysis

The Statistics Package for the Social Sciences (SPSS, version 21; IBM North America, New York, United States) was used for conducting statistical analysis. All the parameters, including interface pressure measurements (TCA_BACK_, AP_BACK_, PP_BACK_, TCA_SEAT_, AP_SEAT_, and PP_SEAT_) and subjective discomfort evaluations (upper-back, mid-back, lower-back, buttocks, and thighs) were compared between the different sitting postures (RSS, FBS, PLS, and BTS) by using a Friedman test. A Wilcoxon signed-rank test was used for detecting statistically significant differences in the dependent variables across the tests. The statistical significance was set at *P* < 0.05.

## Results

Eighteen elderly men were recruited (age, 69.94 ± 3.83 years old; weight, 69.71 ± 10.86 kg; height, 166.10 ± 5.94 cm; body mass index, 25.21 ± 3.43 kg/m^2^). All the participants completed the interface pressure measurements and subjective discomfort evaluations using the experimental wheelchair in the RSS, FBS, PLS, and BTS. No participants reported adverse reactions to the experimental process.

### Interface pressure

**Figure 4:**
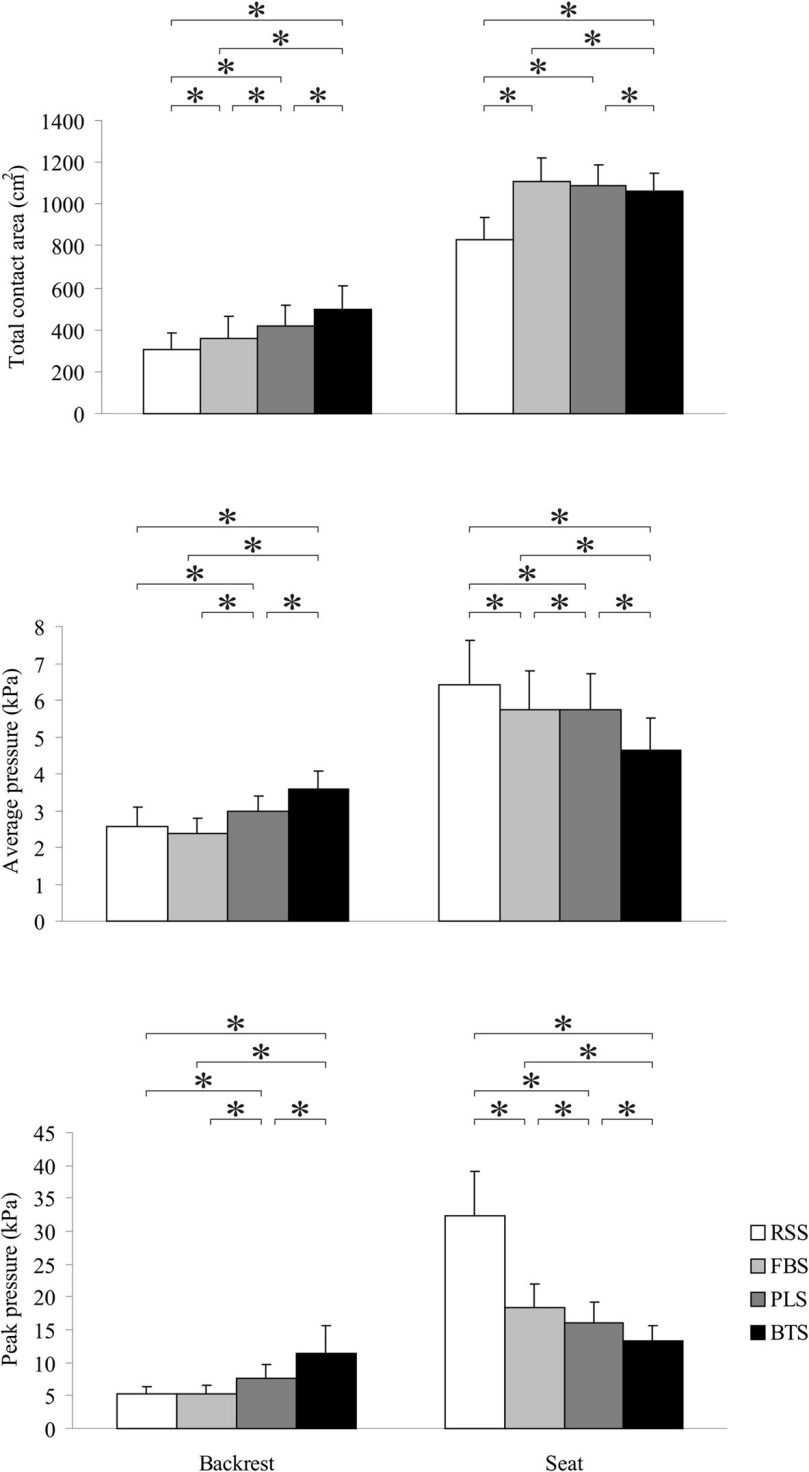
**Results of interface pressure measurements.** Comparison of mean total contact area, average pressure, and peak pressure on both the whole backrest and the entire seat across 4 sitting postures, which include relaxed slouching sitting (RSS), flat back support sitting (FBS), prominent lumbar support sitting (PLS), and backward thoracic support sitting (BTS). Error bars indicate SD and *indicates p < 0.05.

The results of the interface pressure measurements are shown in Figure 4. The outcomes showed that the RSS exhibited the worst overall performance in which AP_SEAT_ and PP_SEAT_ were comparatively high. The BTS demonstrated optimal overall performance in which AP_SEAT_ and PP_SEAT_ were comparatively low and TCA_BACK_, AP_BACK_, and PP_BACK_ were comparatively high. In addition, The TCA_BACK_, AP_BACK_, and PP_BACK_ of BTS (495.97 ± 116.29 cm^2^, 3.59 ± 0.49 kPa, and 11.42 ± 4.26 kPa) appeared to be significantly higher (*P* < 0.001) than those of RSS (307.69 ± 79.91 cm^2^, 2.58 ± 0.50 kPa, and 5.18 ± 1.16 kPa), FBS (356.72 ± 109.23 cm^2^, 2.39 ± 0.39 kPa, and 5.21 ± 1.32 kPa), and PLS (417.34 ± 97.16 cm^2^, 2.99 ± 0.42 kPa, and 7.65 ± 2.02 kPa). Regarding TCA_SEAT_, the BTS (1059.60 ± 88.49 cm^2^) appeared to be significantly lower (*P* < 0.016) than FBS (1107.84 ± 111.08 cm^2^) and PLS (1087.41 ± 102.81 cm^2^), but higher than RSS (830.37 ± 104.48 cm^2^). The AP_SEAT_ and PP_SEAT_ of BTS (4.65 ± 0.85 and 13.21 ± 2.44 kPa) appeared to be significantly lower (*P* < 0.001) than those of RSS (6.43 ± 1.19 and 32.38 ± 6.60 kPa), FBS (5.74 ± 1.05 and 18.39 ± 3.51 kPa), and PLS (5.72 ± 0.98 and 16.14 ± 3.06 kPa).

### Subjective discomfort

**Figure 5:**
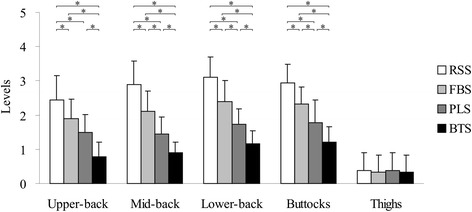
**Results of subjective discomfort evaluations.** Comparison of mean subjective discomfort levels of upper-back, mid-back, lower-back, buttocks, and thighs across 4 sitting postures, which include relaxed slouching sitting (RSS), flat back support sitting (FBS), prominent lumbar support sitting (PLS), and backward thoracic support sitting (BTS). The question items for each body part were evaluated using a scale of 6 levels (0–5) in which 0 represents no discomfort and 5 represents extreme conceivable discomfort. Error bars indicate SD and * indicates p < 0.05.

The results of subjective discomfort evaluations are shown in Figure 5. The outcomes indicated that the RSS exhibited the worst overall performance in which comparatively high subjective discomfort was produced in the back and buttock regions. The BTS demonstrated optimal overall performance in which comparatively minor subjective discomfort was produced in the back and buttock regions. Regarding subjective discomfort of upper-back, mid-back, lower-back, and buttocks, the BTS (0.78 ± 0.43, 0.89 ± 0.32, 1.17 ± 0.38, and 1.22 ± 0.43) appeared to be significantly lower (*P* < 0.008) than RSS (2.44 ± 0.70, 2.89 ± 0.68, 3.11 ± 0.58, and 2.94 ± 0.54), FBS (1.89 ± 0.58, 2.11 ± 0.58, 2.39 ± 0.61, and 2.33 ± 0.49), and PLS (1.50 ± 0.51, 1.44 ± 0.51, 1.72 ± 0.46, and 1.78 ± 0.65); no significant differences in thigh values were observed.

## Discussion

This study investigated and quantified the biomechanical influences of RSS, FBS, PLS, and BTS on interface pressure and subjective discomfort. The results showed that when compared with other sitting postures, BTS can significantly reduce AP_SEAT_ and PP_SEAT_ values, increase AP_BACK_ and PP_BACK_ values, and reduce the subjective discomfort in the back and buttocks. The results indicate that BTS can maintain more comfortable wheelchair sitting postures.

When sitting on ordinary wheelchairs, body weight is primarily supported by the backrest and seat cushion. Particularly, the buttocks support the majority of body weight and stress is concentrated in the IT region and the surrounding soft tissues [27]. Previous studies have indicated that IT stress is closely related to spinal load [5,7,8]. Thus, decreasing IT stress concentrations can reduce the risk of back pain. Our study showed that the PP_SEAT_ values of the 4 sitting postures were included in IT areas; RSS generated significantly higher PP_SEAT_ values than the other sitting postures. Regarding BTS, compared with those of other postures, AP_SEAT_ and PP_SEAT_ values were significantly lower in BTS, whereas the AP_BACK_ and PP_BACK_ values of BTS were significantly higher. These results suggest that a portion of body weight has been shifted from the seat cushion to the backrest. Previous studies have indicated that increased posterior load on backrest, which helps mitigate the stress in the IT area [7,8]. The BTS transferred ischial pressures to the back and reduced the risks of resultant back pain and pressure ulcers [7,8,16,22]. Although the major risk area for pressure ulcers in sitting postures is near the ischial bone, the pressure transferred to the back regions may increase the risk of back pressure ulcers, particularly in the scapula and sacral regions. Therefore, we suggest increasing foam thicknesses in the back region to reduce back pressures.

The musculoskeletal discomfort caused by biomechanical loading may reflect an early perception of pain [10,28]. In particular, discomfort in the lumbar area is the primary factor for an increase in general discomfort in the sitting posture [10,29]. Our study results showed that during the subjective discomfort evaluations, all the participants experienced the highest discomfort in the upper-back, mid-back, lower-back, and buttocks areas in the RSS posture. This can be attributed to the FRP that induces the body weight to generate mechanical loading on passive tissues (ie, intervertebral discs, ligaments, and fascia), which induces creep and stimulates the surrounding sensory nerves, consequently resulting in discomfort [3,4]. The subjective discomfort scores for the upper-back, mid-back, lower-back, and buttocks areas during the BTS posture were the lowest compared with those of the other sitting postures. BTS relieved discomfort because of the following: First, BTS can maintain large degrees of lumbar lordosis and transfer the forces acted on the lumbar vertebrae from the intervertebral discs to the inferior margins of articular surfaces of zygapophysial joints, the influence of intervertebral discs creep is reduced [4,22]. Second, BTS shifts a portion of the body weight and load to the backrest, thereby reducing back muscle load [7].

## Conclusions

We considered the limitations of the human musculoskeletal system in wheelchair sitting postures, proposed an innovative backrest design (ie, backward thoracic support). The results showed that BTS redistributed interface pressure to reduce IT stress concentration and alleviate the subjective discomfort in the back and buttocks, thereby reducing the risk of back pain. Our findings can provide clinical physicians or wheelchair users with a basis for choosing wheelchair seating systems. In this study, we primarily investigated whether BTS effectively reduces the risk of back pain. However, future studies investigating cervical angles and neck and shoulder muscle activities are required to determine whether a BTS-produced reclining upper torso increases the risk of neck pain.
